# Instagram influencers attributes and parasocial relationship: A dataset from Qatar

**DOI:** 10.1016/j.dib.2024.110128

**Published:** 2024-02-01

**Authors:** Sara Al Sulaiti, Mohamed Slim Ben Mimoun, Hatem Elgohary

**Affiliations:** Department of Management and Marketing, College of Business and Economics, Qatar University, University Street, PoBox 2713, Doha, Qatar

**Keywords:** Social media influencer, Parasocial relationship, Purchase intention, WOM, Partial least squares structural equation modeling PLS-SEM

## Abstract

The dataset investigates how social media influencers’ attributes affect followers’ parasocial relationship. It also examines the mediating role of the parasocial relationship between the social media influencers’ attributes and behavioral intentions. A snowballing sampling technique was used to target Instagram users in Qatar. 574 valid responses were analyzed using Partial least squares structural equation modeling (PLS-SEM). The data provides descriptive information about the essential Instagram influencers among users in Qatar. It also gives new insight into the influencers’ characteristics that will impact consumer behavior the most. The dataset could be very helpful for brands and marketers in Qatar in choosing the most effective influencers. The dataset presents a real value for researchers examining social media consumers behavior specifically in GCC countries context or conducting cross-national comparative studies.

Specifications Table

**Table:** 

Subject	Marketing
Specific subject area	Social Media Marketing
Type of data	Table Figure Excel data
How the data were acquired	The data were collected by distributing a Google form link to Instagram users in Qatar. The questionnaire used 5-point Likert versions of well-established measurement scales. Scales content was translated into Arabic, the official language in Qatar. Using a snowball sampling technique, 574 valid answers were collected from Instagram users in Qatar.
Data format	Raw Analyzed Filtered Descriptive data
Description of data collection	The questionnaire included 35 items to measure the main variables (Homophily, Popularity, Leverage, Fashionable, Affinity, WOM, and purchase intentions). We used additional 8 questions to cover the respondents’ profiles. Tables and figures were used to present the data and results covering the descriptive statistics, the quality of the measurement and the structural models, and hypothesis testing.
Data source location	·City/Town/Region: Doha ·Country: Qatar
Data accessibility	Repository name: Mendeley Data (https://data.mendeley.com/) Data identification number: 10.17632/zv6fdn2ysv.3 Direct URL to data: https://data.mendeley.com/datasets/zv6fdn2ysv/3

## 1 Value of the Data

• This dataset is interesting as Qatar is the 3rd (96 %) country in the world regarding the percentage of the population using social networks [1]. In addition, Instagram represents after Facebook, the second most-used social network in Qatar [2].
• The dataset contains measures of the main attributes (i.e., Homophily, Popularity, Leverage, Fashionable, and Affinity) of Instagram influencers in Qatar. The dataset also includes measures of the parasocial relationship between Instagram users and influencers, purchase intention (of product recommended by the social media influencer), and word of mouth (intention to recommend or speak positively about the influencer).
• The dataset includes several control variables (e.g., demographics and usage of Instagram) that may be used to perform more sophisticated analysis as multigroup analysis MGA or test for moderated mediation.
• The dataset could be very helpful for brands and marketers in Qatar in choosing the most effective influencers. Researchers could use the data to identify the fit between the influencers’ attributes and their domain of expertise. Data could also be helpful for future cross-national comparative studies that will replicate the data collection in different cultures, countries, and regions. For future research data could be also coupled with other types of data as data obtained from Data mining [3] or Netnographic data [4].
• Participants in our survey were 38 % males and 61 % females living in Qatar. This distribution could be seen as a limitation of our data, as in Qatar, females represent 27.6 % of the population and 34.6 % of the total Instagram users [5]. However, if we report the percentage of females and males to the number of Instagram users in Qatar (40.7 of the population) and the percentage of Instagram users by sex [5], it is possible to observe a good fit between the distribution of our data and the penetration rate by sex for Instagram users in Qatar: 56 % of females in Qatar use Instagram, and only 35 % of males use Instagram.
• Finally, respondents were 73.3 % Qatari and 26.7 % Non-Qatari Arabic speaking. Qatari citizens represent less than 15 % of the population of Qatar, and Other Arabs represent about 13 % of the total population [6]. However, these segments, specifically the Qatari Citizens, are extremely important from a marketing point of view first because they are one of the wealthiest populations in the world [7] and second because it is difficult to get information from this segment.

## 2 Objective

With the continuous increase in social media networks (SMN) use around the world and the rise of time consumers spend on SMN, it became critical for firms and researchers to understand better how SMN could impact consumers’ behavior and could be used as effective marketing tools. In this context, social media influencers are becoming the “masterpiece” for any effective social media marketing strategy. This dataset investigates in the context of Instagram users in Qatar, the social media influencers’ attributes that effectively impact the parasocial relationship between followers and influencers and lead to behavioral intention: WOM and purchase intentions. It also examines the mediating role of the parasocial relationship between Instagram influencers' attributes and consumers’ behavioral intentions.

In addition, as the dataset includes several control variables (e.g., demographics and usage of Instagram), it would be interesting to explore how the proposed model works differently for different groups of consumers (by sex, age, level of usage of SMN, category of product …).

## 3 Data Description

In total, 900 people received the link to our final questionnaire. 691 responses were collected, indicating a response rate of 76 %. However, only 574 were valid (83 %) and included in our dataset. One hundred seventeen responses (17 %) were eliminated for different reasons, including the age of participants below 18, participants without an Instagram account, or those who do not follow any Instagram influencer.

The dataset associated with this article comes in a raw data table format (.CSV) and an SPSS data file (.sav) that could be used for different analyses. The dataset consists of answers from Instagram users in Qatar asked about their behavior on Instagram, their favorite Instagram influencer, their perceptions of the attributes of their favorite Instagram influencer, their (parasocial) relationship with their favorite Instagram influencer, as well as the possibility of recommending this influencer or purchase goods or services endorsed by their favorite influencer.

A five-point Likert scale survey instrument including 35 items was developed to measure seven different concepts. Considering the importance of the validity and robustness of measurement scales and items, we used only tested and validated scales published in top journals (ranked Q1 CiteScore Best Quartile). Table 1 summarizes the Wording of Measurement Items and their sources. However, after translating the items from English to Arabic and the questionnaire pretest, we slightly changed the wording of some Items to adapt them to the Qatari context and Arabic Language. The changes do not affect the items' meaning nor the scales' content validity. We tested for Construct reliability and convergent validity (Table 5) as well as for discriminant validity (Tables 6 and 7). The results confirm the construct's reliability and validity.

**Table 1:** Wordings of measurement items.

Construct	Description	Sources
Homophily (Hom)	Hom1. This Instagram influencer thinks like me. Hom2. This Instagram influencer is similar to me. Hom3. This Instagram influencer is like me. Hom4. This Instagram influencer shares my values. Hom5. This Instagram influencer has a lot in common with me. Hom6. This Instagram influencer behaves like me. Hom7. This Instagram influencer has thoughts and ideas that are similar to mine. Hom8. I think that my Instagram influencer could be a friend of mine. Hom9. I would like to have a friendly chat with my Instagram influencer.	[9]
Popularity (Pop)	Pop1. This Instagram influencer has a high exposure in the Instagram environment. Pop2. This Instagram influencer has high popularity in the Instagram environment. Pop3. This Instagram influencer has a high reputation in the Instagram environment.	[8]
Leverage (Lev)	Lev1. This Instagram influencer can cause debate in the Instagram environment. Lev2. This Instagram influencer is topical in the Instagram environment. Lev3. This Instagram influencer's remarks in the Instagram environment are sensational.	[8]
Fashionable (Fash)	Fash1. This Instagram influencer can lead the trend in the Instagram environment. Fash2. This Instagram influencer is very fashionable. Fash3. This Instagram influencer is very sensitive to fashion.	[8]
Affinity (Aff)	Aff1. This Instagram influencer is very close to people. Aff2. This Instagram influencer behavior is in a popular style. Aff3. This Instagram influencer is a very down-to-earth person.	[8]
Parasocial relationship (PSI)	PSI1. I feel close enough to my favorite Instagram influencer to use his(her) Instagram. PSI2. I feel comfortable about my favorite Instagram influencer messages. PSI3. I can rely on the information I get from my favorite Instagram influencer. PSI4. I feel fascinated with my favorite Instagram influencer's Instagram. PSI5. In the past, I pitied my favorite Instagram influencer when he/she made a mistake on his/her Instagram. PSI6. I think that my favorite Instagram influencer is helpful for my interests (in fashion and others).	[10]
Word of mouth (Wom)	Wom1. I am likely to say positive things about what my Instagram influencer promotes to others. Wom2. I would recommend what my Instagram influencer promotes to my friends and relatives. Wom3. If my friends were looking for a product or service of this type, I would recommend what my Instagram influencer said about it.	[11]
Intention to Purchase	Int1. I will buy the product or the service that Instagram influencer promoted through Instagram. Int2. I have the intention to buy the product or the service that my Instagram influencer promoted on Instagram. Int3. I am interested in buying the product or the service my Instagram influencer promoted on Instagram. Int4. It is likely that I will buy the products or services my Instagram influencer promoted on Instagram in the future. Int5. Overall, I am pleased with what my Instagram influencer promotes on Instagram.	[12]

**Fig. 1:**
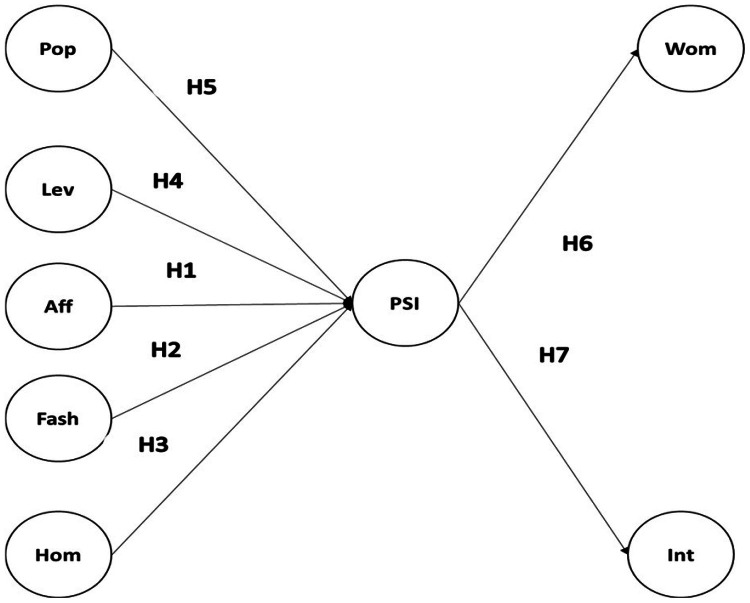
The conceptual model.

For every item, the responses were scored as ‘strongly agree’ 5, ‘agree’ = 4, ‘neutral’ = 3, ‘disagree’ = 2, and ‘strongly disagree’ = 1. First, we adopted four scales from [8] to measure four of the perceived Instagram influencer attributes: Popularity (Pop/ 3 items); Leverage (Lev/ 3 items); Affinity (Aff/ 3 items); and Fashionable (Fash/ 3 items). We measured a fifth Instagram influencer perceived attribute, Homophily (Hom/ 9 items), adapting the scale from [9]. We used an adapted scale from [10] to measure Parasocial relationship (PSI/ 6 items). For behavioral intentions, we used respectively an adapted version of the scale from [11] to measure word of mouth (WOM/ 3 items) and a slightly adjusted version from [12] to measure the intention to purchase (Int/ 5 items). All the scales have reflective Items. Fig. 1 illustrates our conceptual model, and Table 1 represents the wordings of all the items used for the different scales.

Pop=Popularity; Lev=Leverage; Aff=Affinity; Fash=Fashionable; Hom=Homophily PSI=Parasocial relationship; Wom=Word of mouth; Int=intention to purchase.

The dataset also contains different control variables that could be used for additional analysis. We asked the participants about their age, nationality, revenue, and education. Participants were 38 % male and 61 % female living in Qatar. 73.3 % were Qatari, and only 26.7 % were Non-Qatari (Arabic speaking as the survey was in Arabic). The age group was divided into six groups. 65.3 % were between 18 and 24, 24.7 % between 25 and 34, 7.8 % between 35 and 44 years old, 1.7 % between 45 and 54, and only 0.3 % between 55 and 64. The annual income was divided into six groups. Most participants (61.7 %) earn less than QTR 50,000 annually, as most were students. Table 2 summarizes the profile of our sample.

**Table 2:** Profile and demographic characteristics of respondents (*n* = 574).

Attributes	Characteristic	N	Percentage (%)
Sex	Male	220	38.3
	Female	354	61.7
Age	18–24	375	65.3
	25–34	142	24.7
	35–44	45	7.8
	45–54	10	1.7
	55–65	2	0.3
Nationality	Qatari	421	73.3
	Non-Qatari	153	26.7
Annual Income (in QR)	- 50,000	354	61.7
	50,001–150,000	116	20.2
	150,001–250,000	48	8.4
	250,001–350,000	29	5.1
	350,001–450,000	10	1.7
	+450,000	17	3.0

We questioned the participants about their Instagram behavior; we observed that about half of them (49.3 %) spend more than 5 h daily on social media and that the majority of the respondents follow local influencers with substantial diversity in terms of the domain of expertise of their favorite Instagram influencers. Characteristics of respondents related to social media and Instagram behavior are summarized in Table 3.

**Table 3:** Respondents' social media and Instagram behavior (*n* = 574).

Attributes	Characteristic	N	Percentage (%)
Daily Hours spent on SMN	1–2	85	14.8
	3–4	206	35.9
	+5	283	49.3
Favorite Instagram influencer	Abdulla AlGafri	74	12.9
	Haneen AlSaify	27	4.7
	Dr. Mohammed AlSafy	12	2.1
	Noha Nabil	14	2.4
	No signal	15	2.6
	Others	415	72.3
	Cristiano Ronaldo	17	3.0
Number of years following the favorite Instagram influencer	−1Y	80	13.9
	1–2Y	107	18.6
	2–3Y	161	28.0
	+3Y	226	39.4
Area of expertise of the favorite Instagram influencer	Fashion	26	4.5
	Traveling	30	5.2
	Beauty products	16	2.8
	Food and beverages	44	7.7
	Others	272	47.4
	Multiple	186	32.4

Table 4 presents the descriptive statistics for the scales’ items.

**Table 4:** Mean, range, standard deviation, Kurtosis, and Skewness.

Construct	Item	Mean	Range	Standard Deviation	Excess Kurtosis	Skewness
Homophily	Hom1	2.70	4	1.269	−1.002	0.139
	Hom2	2.39	4	1.345	−1.031	0.481
	Hom3	2.22	4	1.276	−0.689	0.661
	Hom4	2.50	4	1.357	−1.076	0.382
	Hom5	3.04	4	1.324	−1.074	−0.049
	Hom6	2.52	4	1.272	−0.968	0.331
	Hom7	3.01	4	1.261	−0.947	−0.053
	Hom8	2.83	4	1.375	−1.158	0.130
	Hom9	2.92	4	1.432	−1.273	0.088
Popularity	Pop1	3.46	4	1.223	−0.800	−0.333
	Pop2	3.60	4	1.230	−0.827	−0.448
	Pop3	3.66	4	1.220	−0.711	−0.507
Leverage	Lev1	3.18	4	1.337	−1.063	−0.142
	Lev2	3.59	4	1.298	−0.862	−0.462
	Lev3	3.40	4	1.249	−0.917	−0.237
Fashionable	Fash1	3.41	4	1.287	−0.967	−0.305
	Fash2	3.42	4	1.228	−0.877	−0.276
	Fash3	3.63	4	1.240	−0.887	−0.430
Affinity	Aff1	3.61	4	1.149	−0.663	−0.391
	Aff2	3.29	4	1.214	−0.804	−0.178
	Aff3	3.75	4	1.166	−0.550	−0.571
Parasocial relationship	PSI1	2.96	4	1.234	−0.865	0.026
	PSI2	3.49	4	1.190	−0.846	−0.281
	PSI3	3.45	4	1.246	−0.881	−0.316
	PSI4	3.29	4	1.281	−0.967	−0.180
	PSI5	2.62	4	1.295	−0.966	0.273
	PSI6	3.34	4	1.209	−0.779	−0.232
Word of mouth	WOM1	3.45	4	1.198	−0.761	−0.286
	WOM2	3.44	4	1.213	−0.739	−0.329
	WOM3	3.30	4	1.243	−0.829	−0.231
Intention to Purchase	Int1	3.00	4	1.199	−0.736	0.068
	Int2	2.95	4	1.206	−0.756	−0.003
	Int3	2.97	4	1.247	−0.818	−0.010
	Int4	3.03	4	1.195	−0.745	−0.106
	Int5	3.33	4	1.199	−0.774	−0.216

We also used the Common Harman's single-factor to assess possible common method variance problems. The results indicate that the variance accounted for in the first factor is 34.6 % lower than 50 %, indicating that the sample did not contain common method bias.

In what follows, we present the PLS-SEM results obtained using SmartPLS.4 [13]. eight tables and two figures summarize the measurement model's quality (the instruments' reliability and validity) and the structural model (correlation and hypothesis testing).

**Fig. 2:**
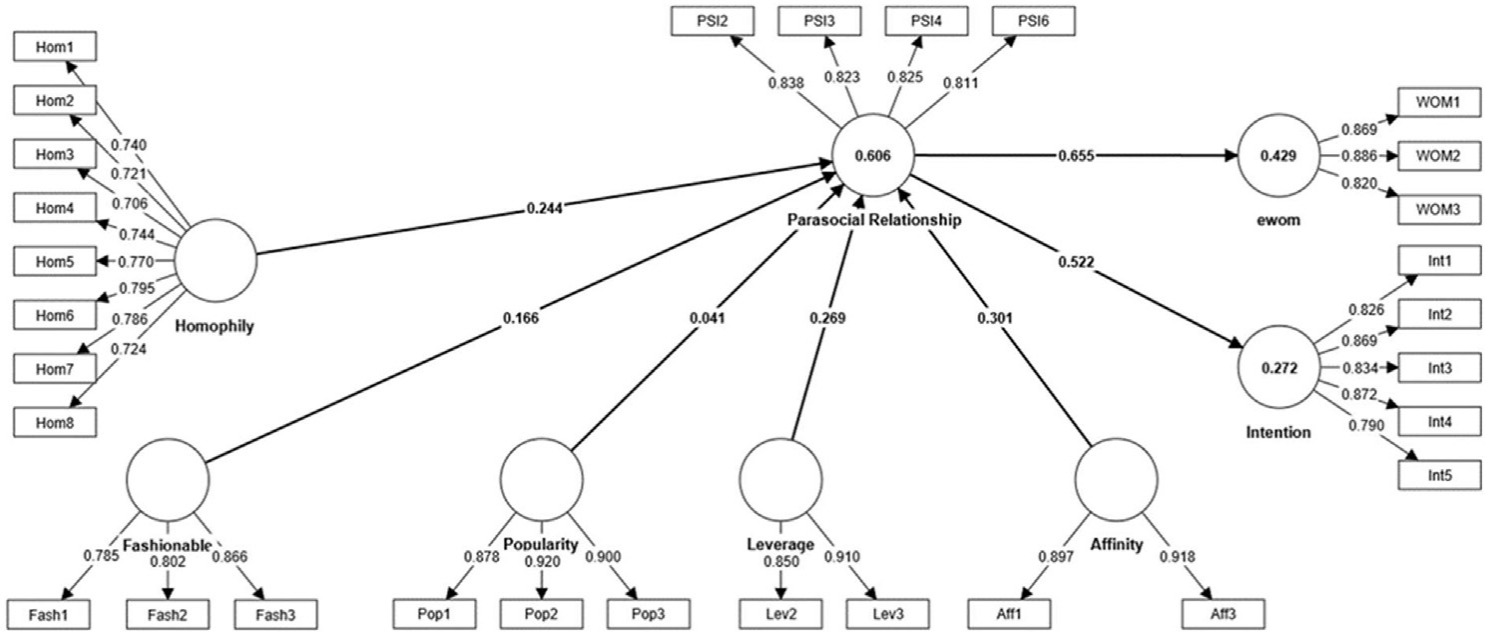
The final measurement model.

Due to low outer loadings, five items were removed from the final measurement model (Hom9, PSI1, PSI5, Lev1, Aff2). The final measurement model is summarized in Fig. 2.

In general, the measurement model indicates good reliability (Table 5), convergent validity (Table 5), and discriminant validity (Tables 6 and 7). All ρA values exceed the standard threshold of 0.7, all average variance extracted (AVE) values are larger than 0.5, and the values of HTMT criterion exceed the conservative value of 0.85 except for one value that is lower than 0.9 and considered acceptable [14].

**Table 5:** Construct reliability and convergent validity.

	Cronbach's alpha	Rho A	Composite reliability (rho_c)	The average variance extracted (AVE)
Affinity	0.788	0.794	0.904	0.824
Fashionable	0.760	0.804	0.859	0.670
Homophily	0.890	0.903	0.911	0.561
Intention	0.896	0.913	0.922	0.704
Leverage	0.713	0.741	0.873	0.775
Parasocial Relationship	0.843	0.843	0.894	0.679
Popularity	0.882	0.882	0.927	0.809
Wom	0.821	0.824	0.894	0.737

**Tables 6:** Discriminant validity Fornell-Larcker criterion.

	Affinity	Fashionable	Homophily	Intention	Leverage	Parasocial Relationship	Popularity	Wom
Affinity	0.908							
Fashionable	0.664	0.818						
Homophily	0.293	0.246	0.749					
Intention	0.361	0.422	0.435	0.839				
Leverage	0.578	0.663	0.262	0.366	0.880			
Parasocial Relationship	0.660	0.626	0.453	0.522	0.641	0.824		
Popularity	0.536	0.545	0.215	0.362	0.590	0.504	0.899	
Wom	0.554	0.525	0.413	0.652	0.530	0.655	0.485	0.858

**Table 7:** Heterotrait-monotrait ratio (HTMT) – Matrix.

	Affinity	Fashionable	Homophily	Intention	Leverage	Parasocial Relationship	Popularity	Wom
Affinity								
Fashionable	0.835							
Homophily	0.320	0.276						
Intention	0.411	0.495	0.485					
Leverage	0.770	0.865	0.307	0.424				
Parasocial Relationship	0.807	0.754	0.496	0.578	0.817			
Popularity	0.644	0.656	0.226	0.388	0.745	0.583		
Wom	0.687	0.644	0.461	0.752	0.685	0.786	0.568	

**Fig. 3:**
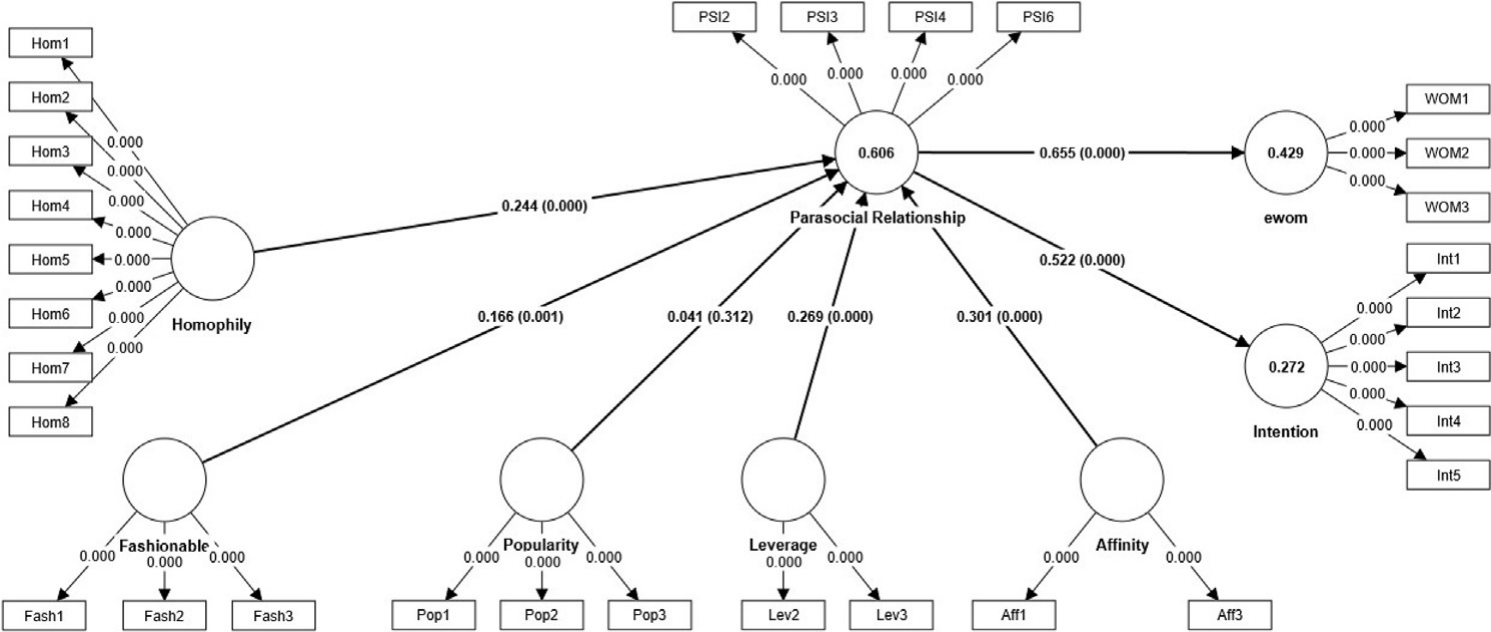
The final structural model.

Fig. 3 and Table 8 summarize the main results considering the structural model.

**Table 8:** Correlation and hypothesis testing.

		Original sample (O)	Sample mean (M)	Standard deviation (STDEV)	T statistics (|O/STDEV|)	P values	Result
H1	Aff-> PSI	0.301	0.301	0.044	6.808	0.000	Supported
H2	Fash-> PSI	0.166	0.167	0.050	3.293	0.001	Supported
H3	Hom-> PSI	0.244	0.245	0.030	8.246	0.000	Supported
H4	Lev -> PSI	0.269	0.268	0.044	6.162	0.000	Supported
H5	Pop -> PSI	0.041	0.041	0.040	1.011	0.312	Not Supported
H6	PSI -> Int	0.522	0.523	0.036	14.610	0.000	Supported
H7	PSI -> Wom	0.655	0.657	0.030	21.575	0.000	Supported

Significant at ρ < 0.05 (5 %).

We also checked that for the Inner model, all the Variance inflation factors (VIF) are lower than 5.00, indicating the absence of high collinearity concerns [12]. Table 9 presents the collinearity test results.

**Table 9:** Inner model - VIF List.

	VIF
Affinity -> Parasocial Relationship	2.033
Fashionable -> Parasocial Relationship	2.308
Homophily -> Parasocial Relationship	1.111
Leverage -> Parasocial Relationship	2.126
Parasocial relationship -> Intention	1.000
Parasocial relationship -> Wom	1.000
Popularity -> Parasocial Relationship	1.714

The results for the coefficient analysis R2 are presented in Table 10 and present, in general, a satisfactory level of explanatory power.

**Table 10:** The coefficient analysis R2.

	R-square	R-square adjusted
Intention	0.272	0.271
Parasocial Relationship	0.606	0.603
wom	0.429	0.428

We tested the predictive power of our model using CVPAT with PLSpredict. As summarized in Tables 11 and 12, the model indicates some predictive power to pass the “naïve” IA benchmark but insufficient predictive power to overcome the more conservative LM benchmark [15].

**Table 11:** CVPAT, PLS-SEM vs. Indicator average (IA).

	Average loss difference	t value	p-value
Intention	−0.237	6.519	0.000
Parasocial Relationship	−0.614	12.151	0.000
wom	−0.450	10.207	0.000
Overall	−0.416	11.438	0.000

**Table 12:** CVPAT, PLS-SEM vs. Linear model (LM).

	Average loss difference	t value	p-value
Intention	0.045	1.710	0.088
Parasocial Relationship	0.005	0.338	0.736
wom	−0.009	0.464	0.643
Overall	0.018	1.269	0.205

We analyzed the model fit using the SRMR as an indicator. The estimated model has an SRMR of 0.09, higher than the 0.08 threshold but would be accepted if we use the less conservative 0.1 threshold.

Finally, as additional indicators of the model fit we calculated the global goodness-of-fit (GoF) criterion and the effect size. We found a GoF of 0.598 higher than the

Threshold of 0.36 confirming the good quality of Fit. Table 13 summarizes the different ƒ² effect size related to the impact of the predictive constructs on the endogenous latent constructs. Results indicate that Parasocial Relationship has a strong explanation power of both Behavioral Intentions and wom.

**Table 13:** ƒ² effect size results.

	ƒ² effect size	Explanation power
Affinity -> Parasocial Relationship	0.113	weak
Fashionable -> Parasocial Relationship	0.030	weak
Homophily -> Parasocial Relationship	0.136	weak
Leverage -> Parasocial Relationship	0.087	weak
Parasocial Relationship -> Intention	0.375	strong
Parasocial Relationship -> wom	0.752	strong
Popularity -> Parasocial Relationship	0.002	

## 4 Experimental Design, Materials and Methods

We used scales adopted from the literature to ensure the constructs' content validity. The data was collected from January 29th to February 16th, 2020, through an online survey using Google-form. As Arabic is the official language in Qatar, the survey was translated into Arabic to ensure higher participation. We used back-translation, the method most frequently adopted and recommended for translating scales from one language to another [16]. After finalizing the Arabic version of the questionnaire, we conducted a pre-test. 10 Instagram users were invited to answer the questionnaire and give their comments.

Consequently, the questionnaire was slightly adjusted. To indicate the time needed to answer the questionnaire, the time each participant took to the pre-testing was measured. The average time was 8.5 min.

The final online questionnaires included four sections. The first section presented the title of the study, its objective, and the time required to complete the questionnaire. It indicates that participation was voluntary and confidential, and participants can withdraw anytime. Finally, it presents the researchers' contact details. The second section measures the respondents' Instagram usage. It consists of four questions: (i.e., daily time spent on social media, the favorite Instagram influencer, number of years following him/her, and favorite Instagram influencer field of expertise). The third section was dedicated to all the measurement items evaluated on a 5-point Likert scale. It consists of 35 items measuring seven constructs. The final and the fourth section asked participants to give personal information, including sex, age, nationality, and annual income.

Participants of the study were Instagram users living in Qatar and aged 18 years old and above.

In the first stage, the questionnaire was distributed to Qatar University students, faculty members, and staff through a broadcast email by the Communications department at Qatar University. More than 25,000 valid emails were sent. However, after two weeks and one remainder, the number of participants was lower than 30, with a response rate lower than 1 %. This meager rate was expected due to the large number of emails sent daily by the Communications department to Qatar University members.

In the second stage, researchers switched to a more effective strategy. A snowball technique was adopted to ensure more answers and a better response rate. 90 Students following the Marketing Research course in the Bachelor program at the College of Business and Economics of Qatar University responded to the questionnaire and sent it to 9 other Instagram users from their families and friends. Students that participated in the data collection received extra credit. We received 691 answers but only 574 valid answers were included in our dataset.

We obtained the descriptive statistics and tested for normality using SPSS28. Normality was checked via the Skewness and Kurtosis indicators. As presented in Table 1, all data presented an appropriate Range of Skewness and Kurtosis values. Skewness values of the data were between −1 and 1, and the Kurtosis values ranged between −2 and 2, indicating that the data is normally distributed.

We used Partial Least Square Structural Equation Modeling PLS-SEM via Smart-PLS4 to evaluate the measurement and structural models. The measurement model indicates good reliability (Table 5), convergent validity (Table 5), and discriminant validity (Tables 6 and 7). Considering the structural model, six out of seven hypotheses were supported, and the model has an adequate explanation power, some predictive power, and an acceptable fit.

## Limitations

As indicated in the “Experimental design, materials, and methods” section, we first sent the questionnaire link to over 25,000 valid emails, but the response rate was lower than 0.2 %. Consequently, we adopted a snowball sampling technique that is effective in situations of difficulty in reaching a specific population or when no population frame is available [17]. Using this technique, we obtained a response rate of 76 % and 574 valid answers, a sample size bigger than the required sample size of 385 (calculated using the Qualtrics sample size calculator [18]). Our sample is also bigger than the samples of all the articles dealing with Instagram consumer behavior in Qatar and published from 2016 to 2022 (see [19]). However, the Snowball technique has limitations as the initial participants select the following members for the sample, creating bias and negatively impacting the sample representativeness [20]. In our situation, we obtained more female than male respondents and a majority of Qataris. As discussed earlier, these two elements could be considered strengths for our dataset but must be considered carefully for future usage of our dataset.

## Ethics Statements

The authors declare that all respondents gave informed consent to participate. All respondents were systematically informed about the study's content and objectives before participation. Participation was entirely voluntary. An IRB exemption letter with research ethics approval number QU-IRB 1195-E/19 was obtained from Qatar University Review Board.

## CRediT authorship contribution statement

**Sara Al Sulaiti:** Conceptualization, Investigation, Methodology. **Mohamed Slim Ben Mimoun:** Conceptualization, Investigation, Methodology, Data curation, Supervision, Validation, Writing – original draft. **Hatem Elgohary:** Data curation, Validation, Writing – review & editing.

## Data Availability

Social media usage in Qatar (Original data) (Mendeley Data).
